# Comparison of functional and patient-reported outcomes following acute, chronic, and nonoperative distal biceps tendon rupture treatments

**DOI:** 10.1016/j.xrrt.2026.100728

**Published:** 2026-03-19

**Authors:** Justen Saini, Laura Morrison, Tomasz Bugajski, Chloe Elliott, Adina Tarcea, Bayan Ghalimah, Alexandra Munn, Kevin A. Hildebrand, Koren E. Roach, Neil J. White

**Affiliations:** a McCaig Institute for Bone and Joint Health, University of Calgary, Calgary, AB, Canada; b Department of Biomedical Engineering, University of Calgary, Calgary, AB, Canada; c Department of Surgery, University of Calgary, Calgary, AB, Canada; d South Campus Research Unit for Bone and Soft Tissue, Calgary, AB, Canada; e Department of Orthopedic Surgery, University of Texas Health Science Center, Houston, TX, USA; f King Abdulaziz University, Jeddah, Saudi Arabia; g Faculty of Kinesiology, University of Calgary, Calgary, AB, Canada; h Department of Radiology, University of Calgary, Calgary, AB, Canada

**Keywords:** Distal biceps tendon rupture, Strength and endurance, Direct repair, Allograft reconstruction, High-flexion angle repair, Nonoperative management

## Abstract

**Background:**

The purpose of this study was to compare functional outcomes of strength and endurance following 4 different treatment options for complete distal biceps tendon (DBT) ruptures: direct repair (DR), allograft reconstruction (AR), high-flexion angle repair (HFA), and non-operative management (NOP).

**Methods:**

A single-center retrospective chart review was performed on all patients with a DBT rupture. Sixty individuals at a minimum of 1-year postinjury participated in this study across the 4 groups: DR (n = 25), AR (n = 9), HFA (n = 11), and NOP (n = 15). Strength and endurance were measured on the injured and contralateral healthy arm using isokinetic dynamometry testing (Biodex Medical Systems, Inc) for forearm supination and elbow flexion. Patient-reported outcomes were collected through the Disabilities of the Arm, Shoulder, and Hand survey, and postsurgery satisfaction levels were collected through a proprietary survey.

**Results:**

There were significant differences in the injury to operation time between surgical groups; DR participants had significantly shorter times (13.8 days) than the HFA (74.9 days, *P* < .001) and AR (208.9 days, *P* < .001) groups.

Within-group comparisons revealed significant differences in flexion strength between arms for the DR (9.9 ± 18.7%; *P* = .003) and NOP groups (16.4 ± 19.6%; *P* = .012), with decreased strength in the injured arm. Significant differences were observed in total work output between arms for the DR, HFA, and NOP groups in supination (DR: 23.8 ± 30.9%, *P* < .001; HFA: 17.6 ± 24.8%, *P* = .020; NOP: 36.8 ± 31.7%, *P* = .001; respectively) and flexion (DR: 12.4 ± 15.6%, *P* < .001; HFA: 10.4 ± 18.4%, *P* = .036; NOP: 17.6 ± 30.1%, *P* = .045; respectively), with the injured arm performing less work.

There were no significant between-group differences in any functional or patient-reported outcomes. While the HFA and DR participants had a significant decrease in work output for the injured arm, they still reported excellent patient-reported outcomes. The NOP group reported the highest mean Disabilities of the Arm, Shoulder, and Hand score (10.90); however, this did not exceed the threshold of the Minimal Clinically Important Difference (10.83).

**Conclusion:**

Surgical repairs generally preserve functional strength, particularly in supination. Anatomic suspensory fixation repairs (DR and HFA) and nonoperative management may lead to reduced endurance capacity in the injured arm. Despite functional differences between the healthy and injured arm, operative and nonoperative treatment for DBT rupture can yield satisfactory outcomes, highlighting the need for prospective randomized trials to determine the optimal treatment for DBT ruptures.

Distal biceps tendon (DBT) ruptures can lead to impairments in the upper extremity, particularly causing weakness in forearm supination and elbow flexion.^40^ These injuries occur at a rate of 2.55 per 100,000 patients, and epidemiological studies have reported an increased incidence of the injury.^22^ This trend can be attributed to the higher physical demands of the middle-aged population and improved diagnosis with clinical tests.^22^^,^^27^ These injuries can lead to delays in returning to work and modification of duties following treatment.^35^

DBT ruptures predominantly occur in the dominant arm of males aged 40-60 years old who work in manual labor or participate in sports.^14^ The common mechanism of injury involves an eccentric load applied to a flexed elbow, resulting in acute pain and loss of strength during supination and flexion.^7^ Because manual labor and sporting tasks require strength in these movements, and repetitive tasks require endurance, strength and endurance are key functional outcomes evaluated following DBT ruptures.

For a complete DBT rupture, operative management is generally recommended for younger and active individuals, as they yield favorable patient-reported outcomes (PROs), pain reduction, and improved function.^4^ However, operative management is associated with several complications, such as nerve injuries, heterotopic ossification, and re-ruptures, leading to complication rates of up to 28%.^1^^,^^2^^,^^8^ Nonoperative management is an alternative for lower-demand patients but can lead to functional limitations, including decreases in strength and endurance.^10^^,^^23^^,^^26^

In acute cases, surgical repair involves débridement of the ruptured DBT and fixation of the tendon to the radial tuberosity.^40^ This direct repair (DR) may be secured using suspensory fixation such as an EndoButton through a transosseous tunnel, interference fixation, or a suture anchor.^3^,^39^ These DR techniques have demonstrated good strength recovery in flexion and supination compared to conservative treatments.^23^

Delayed diagnosis or treatment can lead to chronic DBT ruptures, defined as cases diagnosed more than 6 weeks post-injury. For these cases, tissue atrophy, significant DBT retraction, and tissue scarring increase the technical demands of the surgical repair and the likelihood of complications.^19^ The time from injury to surgery will impact surgical decision-making; DR is the standard for acute cases but is not always feasible for chronic injuries.^29^ For chronic cases, a tendon graft is often employed to restore tendon length and allow for reattachment of the distal tendon to the radial tuberosity. Several graft techniques, including autografts and allografts, can be used for these chronic injuries to assist with tendon reattachment.^18^^,^^21^^,^^36^ However, these techniques have shown deficits in flexion and supination strength in the injured arm compared to the unaffected arm.^15^ A recently adopted technique used to treat chronic tears is a high-flexion angle repair (HFA), where the tendon is reattached in 60-100° of elbow flexion, with the arm extension angle gradually increased during recovery.^28^ Despite increased clinical use, limited data exist on the functional outcomes associated with HFA, and the preferred treatment for chronic DBT ruptures is not well established.

The purpose of this study was to compare the functional outcomes and PROs of 4 treatment options for complete DBT ruptures: DR, allograft reconstruction (AR), HFA, and nonoperative management. A secondary purpose of this study was to determine if there were differences in functional outcomes depending on if the dominant or nondominant arm was injured. We hypothesized that the injured arm would experience significant decreases in strength and endurance relative to the contralateral uninjured arm. Additionally, we hypothesized that the HFA group would have similar strength and endurance deficits to the other 2 surgical treatments, and the nonoperative group would present the largest deficits in injured arm strength and endurance.

## Materials and methods

A single-center retrospective chart review of medical records from 2012 to 2022 was performed to identify individuals treated for a DBT rupture. All patients treated at this center during this time period were males. This study was conducted under institutional approval by the Conjoint Health Research Ethics Board at our institution (REB21-1851). Patients with a clinically or imaging-confirmed complete distal biceps rupture, treated at least one year prior, were recruited. Participants were excluded if they had a contralateral or ipsilateral arm injury or a condition that impacted their arm strength, endurance, or range of motion (ROM). All participants provided informed consent before taking part in the study.

**Figure 1:**
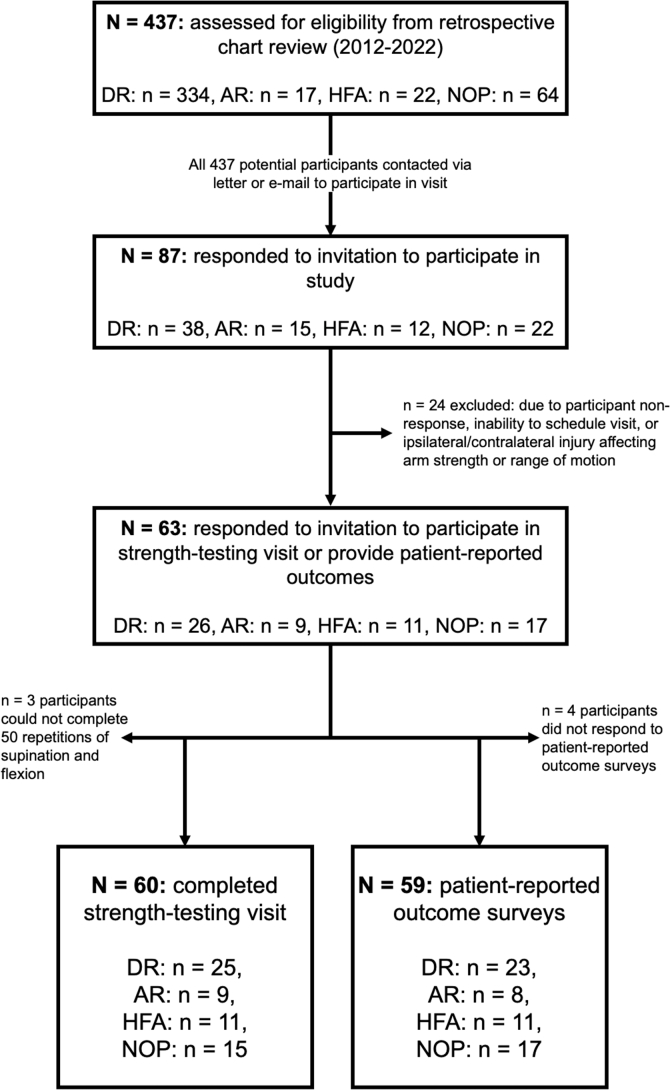
Workflow diagram detailing number of participants involved in this study, spanning from the retrospective chart review to the number of participants who participated in the strength testing visit and the patient-reported outcome surveys. *DR*, direct repair; *AR*, allograft reconstruction; *HFA*, high-flexion angle repair; *NOP*, non-operative management.

Sixty individuals (60 males, 0 female) consented to participate in a strength testing visit (Fig. 1). These participants were categorized into 4 different treatment groups: 25 individuals underwent a DR, 9 underwent AR, 11 underwent HFA, and 15 were managed treated with non-operative management. An additional 3 participants (1 DR, 2 NOP) submitted responses for PRO surveys but did not participate in the strength testing visit.

Strength testing consisted of a single visit to assess long-term strength and endurance outcomes. Participant demographics (age, height, weight, and work status), medical history, mechanism of injury information, active elbow ROM, and strength and endurance measurements in supination and flexion were collected during this session. Two self-reported questionnaires were completed: the Disabilities of the Arm, Shoulder, and Hand (DASH) survey^17^ and a proprietary Biceps Questionnaire (Appendix A) to assess participant satisfaction levels and motivation for having surgery. The occupational data for the participants in the strength testing visit, categorized as active, sedentary, or retired/unemployed, were recorded (Appendix B).

### Surgery descriptions

The technique used for patients is dependent on the time from injury to surgery and the quality and amount of retraction of the tendon, following standard clinical practice.^29^ DR was reserved for acute tears, whereas for chronic tears, HFA was selected when the tendon could be reattached in elbow flexion, whereas AR was used when tendon retraction did not allow for reattachment in flexion. NOP was offered to lower-demand patients or those who opted out of surgery.

DR: A single-incision technique was used to restore the native biceps tendon to the radial tuberosity. The tendon was secured using suspensory fixation similar to the technique described by Dillon et al.^12^ Some surgeons added an interference screw for additional fixation.

AR: The AR technique was determined by the treating surgeon at the time of the operation. All allografts were either hamstring tendon or Achilles tendon. A transosseous EndoButton was used as the fixation method.^32^

HFA: The HFA repair technique was identical to the DR technique, with the exception that the surgeon had to flex the elbow greater than 60° to restore the tendon to the radial tuberosity. Following surgery, the elbow is immobilized in the flexed position in a brace, with gradual increases in extension for up to 8 weeks.^28^

### Dynamometry data acquisition

**Figure 2:**
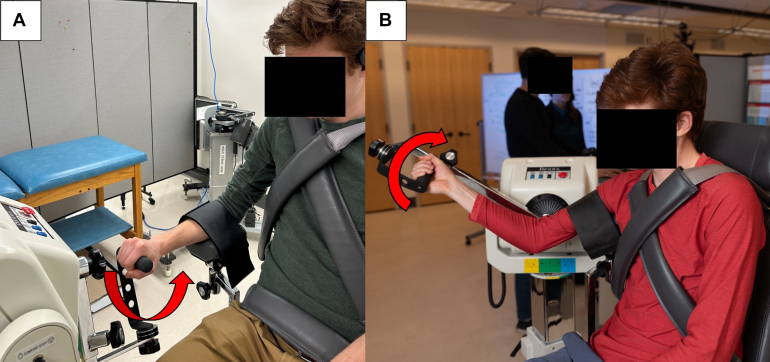
Dynamometer setup configurations for (**A**) pronation-supination, and (**B**) flexion-extension. Participants were securely positioned in the system with straps across the torso to prevent excessive torso and shoulder motion.

To assess strength and endurance, isokinetic testing at 120°/second was performed on a dynamometer (Biodex System 4 Pro™, Biodex Medical Systems, Inc., Shirley, NY). Participants were securely positioned in the system with straps to prevent excessive torso and shoulder motion. Testing included bilateral forearm supination/pronation followed by elbow flexion/extension, following the isokinetic dynamometry protocol for these movements described by Redmond et al.^34^ Briefly, the dynamometer was set up for forearm pronation/supination such that the axis of rotation was aligned with the head of the radius proximally and the head of the ulna distally. For elbow flexion/extension, the axis of rotation was aligned with the trochlea and the capitulum of the humerus (Fig. 2). The strength testing was performed by 3 investigators (J.S., T.B., and C.E.).

The protocol consisted of a 4-repetition warmup, followed by a 1-minute rest before performing a 50-repetition trial. participants were instructed to perform at maximum effort during the concentric motion of the biceps (forearm supination and elbow flexion) and to perform minimal effort during the eccentric motion (forearm pronation and elbow extension). In line with previous DBT functional testing,^34^ exercises were completed first on the healthy (contralateral) arm to establish baseline strength and endurance capacity and then repeated on the injured arm.

Three measurements were obtained during dynamometry testing. Strength was assessed with peak torque, measured as the maximum value of torque generated during the 50 repetitions. Endurance was assessed using total work and work fatigue. Total work was the total amount of work performed over the 50 repetitions, and work fatigue was calculated as the percentage difference in work performed between the first third of testing (first 16 repetitions) and the final third of testing (final 16 repetitions).^11^^,^^34^

### Statistical analysis

To assess if the injured arm exhibited differences in strength and endurance relative to the contralateral arm, paired Student's *t*-tests comparing injured and contralateral arm measurements were employed. Active elbow ROM was measured bilaterally for forearm pronation/supination and elbow flexion/extension using a goniometer; within-group differences were measured as the difference between the contralateral and injured arms.

To evaluate between-group differences in strength and endurance deficits, a multivariate analysis of covariance was conducted using the percentage deficit in the injured arm output compared to the contralateral arm's output. These percentage deficits were evaluated for peak torque and total work as the dependent variables, with arm dominance as a covariate (ie, if the injured arm was the participant's dominant arm). Post hoc pairwise comparisons were performed using Tukey's Honestly Significant Difference test to assess significant differences between treatment groups. Between-group comparisons of the attrition rates (ie, determining if certain groups were not able to complete 50 repetitions of a movement) were evaluated using a Fisher's exact test. To determine if certain treatment groups exhibited better subjective outcomes than others, between-group differences in DASH scores were assessed with a one-way analysis of variance, with a post hoc Tukey's Honestly Significant Difference test to assess significant differences. Statistical significance was set at *P* < .05.

Patient levels of satisfaction following surgery were recorded from the proprietary Biceps Questionnaire (DR, AR, and HFA groups). Participants were prompted to answer satisfied, somewhat satisfied, or not satisfied for prompts regarding their arm health and yes/no to questions regarding how their injury impacts their daily activities. Participants ranked the importance of different reasons for getting surgery from 1 to 5, with 1 being less important and 5 being most important.

A post hoc power analysis was performed using observed effect sizes (Cohen's *d* for within-group comparisons, Cohen's *f* for between-group comparisons) to estimate the achieved power for the study's strength, endurance, and DASH score outcomes.

## Results

### Participant demographics

Demographic data for the 60 participants who attended the strength testing visit are presented in Table I. Time from injury to operation differed significantly between groups, being shorter in DR participants compared to AR (*P* < .001) and HFA (*P* < .001) participants.

**Table I:** Demographic information for participants that attended strength testing visit, reported as mean (SD).

Group	Direct repair	Allograft reconstruction	High-flexion angle repair	Nonoperative management	Total
# of participants	25	9	11	15	60
Age (yr)	50.3 (8.6)	52.9 (7.4)	50.3 (9.3)	54.3 (10.4)	51.7 (9.0)
Body mass index (BMI) mean (SD)	30.4 (5.5)	29.6 (3.9)	30.8 (4.3)	30.2 (3.9)	30.3 (4.6)
Time since injury (mo) mean (SD)	58.0 (27.0)	72.6 (23.7)	54.1 (23.4)	44.2 (29.4)	56.0 (27.4)
Time since operation (mo) mean (SD)	57.5 (26.9)	65.7 (17.2)	51.7 (22.8)	-	57.7 (24.3)
Injury to operation time (d) mean (SD)	13.8 (13.3)	208.9 (274.4)	74.9 (119.5)	-	67.8 (150.9)
Left/right arm	20/5	4/5	5/6	5/10	34/26
Dominant/nondominant arm	8/17	5/4	6/5	9/6	28/32

*SD*, standard deviation.

Five participants (1 AR, 4 NOP) could not complete 50 repetitions of supination due to discomfort in their arms and were not included in the analysis of functional measurements. This attrition rate in the NOP group was significantly higher than in the DR group (*P* = .015). Two NOP participants could not complete the 50 repetitions of flexion due to discomfort in their arms and were not included in the analysis of functional measurements.

### Within-group comparisons (injured-contralateral arm comparisons)

#### Supination

**Figure 3:**
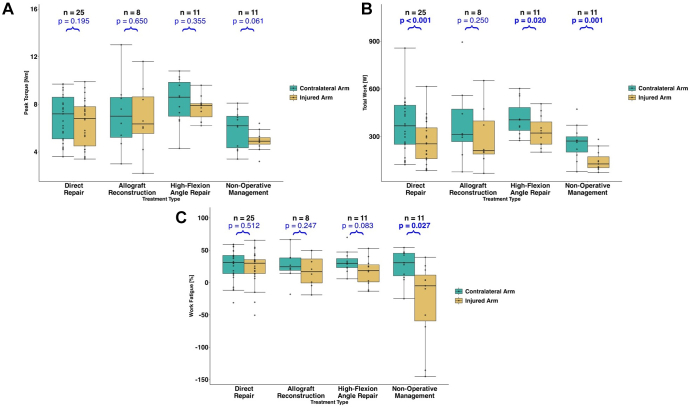
Supination strength and endurance measurements, with the contralateral arm denoted in green and the injured arm denoted in yellow for (**A**) peak torque, (**B**) total work, and (**C**) work fatigue. *P* values are for within-group comparisons of the injured and contralateral arms. Strength was assessed with peak torque. Endurance was assessed using total work (performed over the 50 repetitions) and work fatigue (differences in work performed between the first and final third of testing).

There were no significant within-group differences in supination peak torque between arms for any group (Fig. 3*A*). The DR (*P* < .001), HFA (*P* = .020), and NOP (*P* = .001) groups performed significantly less supination total work in their injured arm compared to their contralateral arm (Fig. 3*B*). The NOP group exhibited significantly less (*P* = .027) work fatigue in their injured arm compared to their contralateral arm (Fig. 3*C*).

#### Flexion

**Figure 4:**
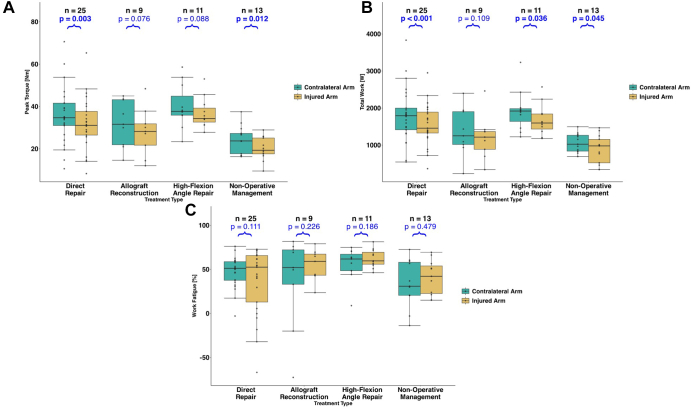
Flexion strength and endurance measurements, with the contralateral arm denoted in green and the injured arm denoted in yellow for (**A**) peak torque, (**B**) total work, and (**C**) work fatigue. *P* values are for within-group comparisons of the injured and contralateral arms. Strength was assessed with peak torque. Endurance was assessed using total work (performed over the 50 repetitions) and work fatigue (differences in work performed between the first and final third of testing).

Flexion peak torque was significantly lower in the injured arm for the DR (*P* = .003) and NOP (*P* = .012) groups (Fig. 4*A*). Flexion total work was significantly less in the injured arm for the DR (*P* < .001), HFA (*P* = .036), and NOP (*P* = .045) groups (Fig. 4*B*). There were no significant differences in work fatigue between arms for any group (Fig. 4*C*).

#### Limb dominance

Participants who injured their nondominant arm had significantly greater deficits in supination peak torque (*P* < .001) and total work (*P* < .001) than participants with dominant-arm injuries (Table II).

**Table II:** Comparison of deficits in the injured arm with respect to the contralateral arm for supination and flexion, categorized by arm dominance.

Movement	Group	Affected dominant arm	Affected nondominant arm	Dominant – nondominant comparison *P*
Supination	Peak torque % deficit mean (SD)	−6.2 (20.4)	14.8 (22.6)	**<0.001**
	Total work % deficit mean (SD)	6.4 (30.2)	39.0 (21.7)	**<0.001**
Flexion	Peak torque % deficit mean (SD)	7.6 (18.3)	14.0 (17.6)	0.092
	Total work % deficit mean (SD)	3.6 (21.6)	20.1 (17.5)	**0.001**

Bolded values indicate significant differences (*P* < 0.05) between dominant and nondominant arms.

Participants who injured their nondominant arm had significantly larger deficits in flexion total work (*P* = .001) than participants with dominant-arm injuries. Participants with nondominant arm injuries had a higher mean deficit than participants with dominant arm injuries; however, this difference was not significant (*P* = .092).

### Between-group comparisons

#### Supination

There were no significant between-group differences in supination peak torque and total work deficits between groups (Table III). The largest mean loss for both deficit measurements was in the NOP group.

**Table III:** Comparison of supination deficits in the injured arm with respect to the contralateral arm, categorized by treatment group.

Group	Peak torque % deficit mean (SD)	Total work % deficit mean (SD)
Direct repair	4.4 (23.5)	23.8 (30.9)
Allograft reconstruction	4.0 (26.2)	17.4 (34.0)
High-flexion angle repair	1.9 (25.0)	17.6 (24.8)
Nonoperative management	11.6 (24.2)	36.8 (31.7)

#### Flexion

There were no significant differences in flexion peak torque or total work deficits in the injured arm between groups (Table IV).

**Table IV:** Comparison of flexion deficits in the injured arm with respect to the contralateral arm, categorized by treatment group.

Group	Peak torque % deficit mean (SD)	Total work % deficit mean (SD)
Direct repair	9.9 (18.7)	12.4 (15.6)
Allograft reconstruction	12.3 (19.7)	5.4 (23.6)
High-flexion angle repair	5.8 (13.7)	10.4 (18.4)
Nonoperative management	16.4 (19.6)	17.6 (30.1)

#### Range of motion

There were no significant differences in ROM between arms for pronation-supination or flexion-extension (Table V).

**Table V:** Pronation-supination and flexion-extension range of motion difference measurements.

Group	Pronation-supination range of motion difference [°] Mean (SD)	Flexion-extension range of motion difference [°] Mean (SD)
Direct repair	1.3 (9.6)	0.2 (5.3)
Allograft reconstruction	2.4 (7.6)	−2.7 (9.8)
High-flexion angle repair	−7.1 (12.4)	−0.9 (9.7)
Nonoperative management	2.8 (7.5)	2.6 (9.8)

#### Patient-reported outcomes

Four participants (3 DR, 1 AR) participated in the strength testing but did not complete the PRO surveys. Three participants (1 DR, 2 NOP) did not complete strength testing but completed the PRO surveys, bringing the total number of DASH survey responses to 59.

**Figure 5:**
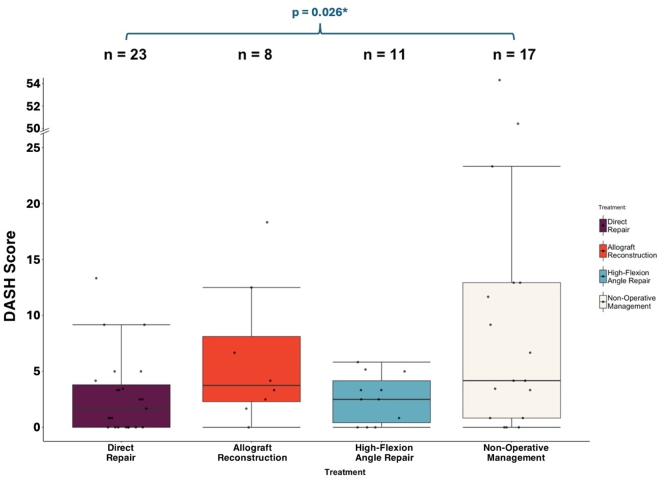
Boxplot comparisons of the DASH scores for the 4 treatment groups. Lower scores indicate lower levels of limb dysfunction. *DASH*, Disabilities of the Arm, Shoulder, and Hand.

The NOP group reported significantly greater DASH scores (10.90 ± 14.87) compared to the DR group (2.83 ± 3.57, *P* = .026). There were no significant differences in DASH scores between any other groups, with the AR group (DASH score = 6.15 ± 6.23) and HFA group (DASH score = 2.59 ± 2.17) presenting acceptable subjective outcomes (Fig. 5).

**Figure 6:**
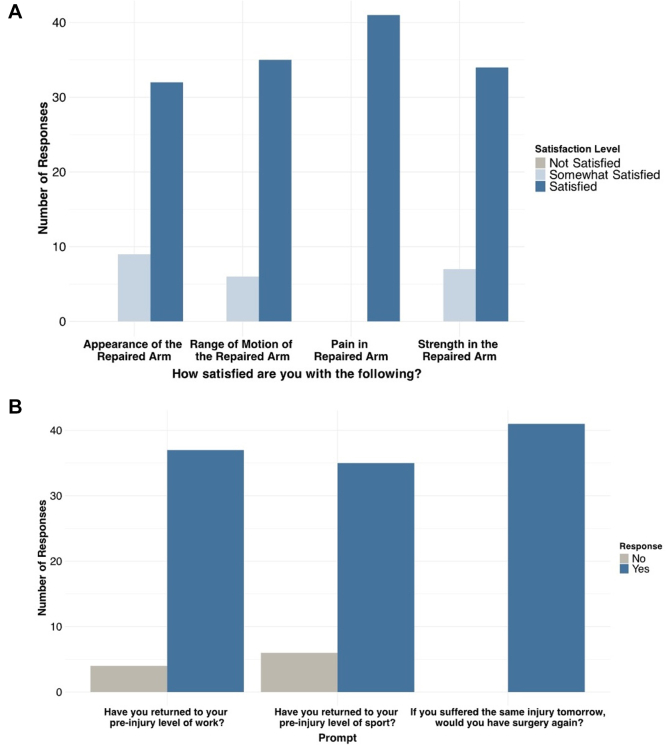
Patient-reported levels of postsurgery satisfaction: (**A**) patient levels of satisfaction for 4 different prompts regarding arm health, and (**B**) patient levels of satisfaction for 3 different prompts regarding how undergoing surgery impacts their daily life.

All postsurgery participants indicated satisfaction or mild satisfaction with all outcomes of their surgery (Fig. 6*A*), and all stated that they would choose surgery again under similar circumstances (Fig. 6*B*).

**Figure 7:**
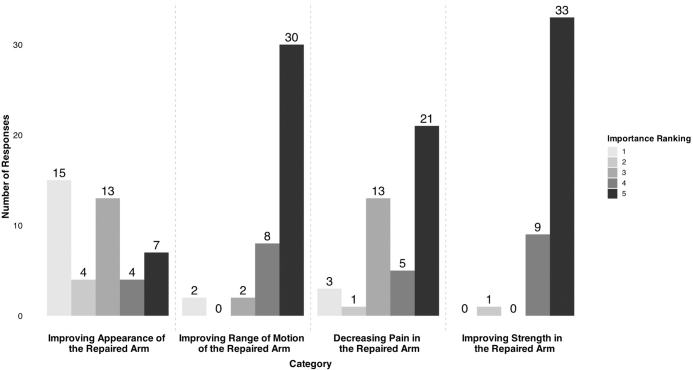
Ranked importance ratings for participants who underwent surgery. All participants ranked the importance for each prompt from 1 to 5, with 1 ranked as low importance and 5 ranked as high importance.

The high-importance reasons for getting surgery were to improve the strength of the repaired arm (33 participants with a 5/5 rating) and to improve the ROM of the repaired arm (30 participants with a 5/5 rating) (Fig. 7).

#### Post hoc power analysis

Post hoc power analysis showed that within-group endurance outcomes achieved moderate to high power, whereas strength outcomes demonstrated lower and more variable power (0.07-0.88) (Table VI). Between-group effect sizes for functional outcomes were small, resulting in low achieved power (0.12-0.26), while the DASH score demonstrated a moderate effect size and a correspondingly high power (Table VII).

**Table VI:** The effect size and power determined by a post hoc analysis for the within-group comparisons of functional outcomes (peak torque and total work in supination and flexion) for the 4 treatment options.

Measure	Direct repair (DR)		Allograft reconstruction (AR)		High-flexion angle repair (HFA)		Nonoperative management (NOP)	
	Effect size (*d*_*z*_)	Power (1 - β)	Effect size (*d*_*z*_)	Power (1 - β)	Effect size (*d*_*z*_)	Power (1 - β)	Effect size (*d*_*z*_)	Power (1 - β)
Supination peak torque	0.27	0.25	0.17	0.07	0.29	0.14	0.64	0.48
Supination total work	0.76	0.95	0.44	0.19	0.84	0.71	1.38	0.98
Flexion peak torque	0.66	0.88	0.68	0.43	0.57	0.40	0.82	0.78
Flexion total work	0.96	0.99	0.60	0.35	0.73	0.59	0.62	0.54

**Table VII:** The effect size and power determined by a post hoc analysis for the between-group comparisons of functional outcomes (peak torque and total work in supination and flexion) and patient-reported outcomes (DASH score).

Measure	Cohen's *f*	Power (1 - β)
Supination peak torque	0.14	0.12
Supination total work	0.23	0.26
Flexion peak torque	0.20	0.21
Flexion total work	0.18	0.18
DASH score	0.43	0.76

*DASH*, Disabilities of the Arm, Shoulder, and Hand.

## Discussion

This study evaluated functional outcomes of strength and endurance between arms and between treatment groups, as well as PROs between groups following a DBT rupture. Our results highlight that there are measurable differences in strength and endurance in the injured arm compared to the contralateral arm, but these deficits did not differ significantly between treatment groups. Contrary to our hypothesis, nonoperative participants did not experience significantly worse functional outcomes compared to surgical treatment groups and reported minimal dysfunction in PRO surveys. These findings support individualized treatment plans, as both operative and nonoperative management can provide acceptable outcomes.

### Within-group comparisons (injured-contralateral arm comparisons)

Strength differences between arms following a DBT rupture varied depending on the type of motion evaluated. While there were no significant within-group peak torque differences between the injured and contralateral arms in supination, there were significant differences in flexion peak torque between arms in the DR and NOP participants. The decrease in DR flexion strength in the injured arm was particularly interesting, as previous studies have found that surgical repairs will adequately restore flexion strength.^16^^,^^25^ This discrepancy may be attributed to the suspensory fixation technique used for DR participants in this study; previous studies suggest that footprint-based DRs can lead to improved function in comparison to suspensory fixation.^6^^,^^31^ This suspensory-based deficit may be due to the anterior, non-anatomic reattachment point used in the Endobutton procedure; this reattachment point has been shown to decrease the moment arm of the DBT.^37^^,^^38^ In contrast, footprint-based repairs aim to restore the native tendon footprint, which may replicate native biomechanical performance.^6^ While additional investigation is required, broader clinical adoption of footprint-based repairs may help decrease the strength difference between arms currently observed with suspensory fixation DR patients.

Across treatment groups, injured arms demonstrated significantly reduced endurance capacity compared to the contralateral arm. Supination and flexion total work were significantly lower in the injured arm of the DR, HFA, and NOP groups. This contrasts with previous findings that surgical repairs will restore near-normal supination and flexion endurance capacity.^11^^,^^26^ These contrasting findings to previous studies may also be due to the suspensory fixation technique used with DR and HFA patients. However, this difference may also be attributed to the different protocols across the studies, which include shorter trials and faster dynamometer speeds. No significant differences were observed between arms for participants with AR, likely due to the small sample size in this group. Overall, these findings emphasize the importance of improving supination endurance during DBT rupture rehabilitation to help address these decreases in total work output in the injured arm.

Comparison of injured and contralateral arm fatiguability highlighted contrasting responses to repetitive motion in supination and flexion. In supination, the only significant difference between arms in work fatigue was in the NOP participants, who demonstrated significantly less work fatigue in the injured arm. This may not reflect improved endurance, which would be reflected in a corresponding increase in total work output. Instead, this behavior suggests consistently reduced torque generation through the repetitive supination task; this trend was also observed by Nesterenko et al with untreated DBT ruptures.^30^ Clinically, this suggests that NOP supination endurance deficits may stem from reduced baseline force generation, potentially due to persistent weakness or altered muscle recruitment, rather than an inability to resist fatigue. This contrasts with the behavior observed in flexion fatigue, where there was no statistically significant difference between arms across all groups. The positive work fatigue values suggest a progressive decline in work output in the final third of repetitions and an inability to sustain maximal torque output throughout the activity. Accordingly, flexion endurance deficits following a DBT rupture can be attributed to fatigue rather than reduced strength. Clinically, this highlights the need for rehabilitation strategies aimed at sustaining flexion force outputs during repetitive motion.

### Limb dominance findings

Limb dominance significantly impacted the deficits observed in strength and endurance, which may have contributed to the deficits observed in the DR group. Only 32% of the DR group had dominant arm injuries, and these dominant-arm injuries presented smaller deficits compared to participants with nondominant arm injuries. Across all groups in supination, there was no deficit observed when the injured arm was the participant's dominant arm, and on average, the injured arm had higher peak torques than the contralateral arm. This characteristic with dominant-arm injuries may reflect more routine use of the dominant arm in common tasks that require supination, such as turning a key or using a screwdriver. This may lead to a natural asymmetry in supination strength between arms, ultimately improving the strength and subsequent recovery in the dominant arm. Greater mean deficits across all groups in supination and flexion endurance were observed with non-dominant arm injuries, consistent with previous findings that nondominant DBT injuries can lead to deficits in endurance capacity.^24^^,^^34^ For these nondominant arm injuries, more aggressive rehabilitation may be required to fully restore functional endurance, where routine supination usage (eg, turning a doorknob or using a screwdriver) may not be as common.

### Between-group comparisons

The HFA group did not exhibit significantly different strength or endurance deficits compared to the other surgical treatments, which supports our original hypothesis that HFA would provide similar functional outcomes to the other surgical groups. Furthermore, this group reported favorable DASH scores that were in alignment with the other surgical groups and high levels of postsurgery satisfaction, indicating satisfactory outcomes and low dysfunction during everyday activities. These outcomes suggest that HFA is a viable option for chronic DBT ruptures where DR is not feasible and significant tendon retraction has occurred.

NOP functional outcomes did not significantly differ from the 3 surgical groups, which did not support our original hypothesis that the largest deficits would be observed in this group. However, across all functional deficit measurements, the NOP group demonstrated the largest mean deficits in their injured arm. The attrition rate of the NOP participants was also significantly higher than the DR group in supination, which may indicate that functional performance, specifically a higher susceptibility to supination fatigue, may be affected with this treatment option. These functional deficits in the NOP group highlight the importance of setting patient expectations when providing personalized treatment plans, and these potential limitations should be communicated to individuals that elect for this treatment. Despite these trends in functional deficits and high attrition rates, NOP patients still reported acceptable treatment outcomes. Although the NOP group had the highest self-reported levels of dysfunction, this was not significantly different with respect to other group means and did not exceed the Minimal Clinically Important Difference for upper-extremity injuries (Minimal Clinically Important Difference = 10.83).^13^ These self-reported results reinforce that both operative and nonoperative options can yield satisfactory recovery, underscoring the importance of individualized treatment.

The findings of this study suggest that both operative and nonoperative treatments can result in good patient-reported and functional outcomes following DBT rupture, with some functional deficits observed regardless of the treatment. While the post hoc power analysis indicates that this study was not powered to detect small differences across treatment groups, the results still provide valuable insight into the functional patterns present in the cohort that can be further analyzed in future studies. The lack of statistically significant between-group functional differences, combined with the low achieved power, suggests subtle treatment differences may exist but remain undetected. These trends highlight the need for larger, prospective studies and align with recent literature aimed at identifying the best treatment for DBT ruptures between operative and non-operative management.^5^^,^^26^^,^^33^ However, most studies to date have been limited to retrospective studies,^9^^,^^20^^,^^23^ and there remains a lack of high-level evidence to support surgical repair over non-operative management. This study highlights the difficulty that clinicians experience in identifying the optimal treatment for a DBT rupture and the need for further examination of different treatment options.

### Limitations

There were limitations to this study that warrant consideration. First, a larger sample size is needed to further examine these results. Despite having one of the largest cohorts of strength and endurance measurements following a DBT tear in literature, this study was underpowered, preventing subgroup analyses based on factors such as age, arm dominance, and occupation. Additionally, as a retrospective study, we did not standardize rehabilitation protocols or account for variations in time between injury and treatment or time between treatment and strength testing visit. There was also a lack of heterogeneity in surgical approach and surgeons across cohorts, which may affect the generalizability of our results. The increased attrition rate of the NOP participants must also be considered when evaluating functional outcomes. As some NOP participants could not complete the 50 repetitions and were excluded from functional deficit analyses, results of the NOP group are biased towards the functionally more fatigue-resistant individuals of this cohort. There was a lack of pre-operative functional and PROs, so we could not assess longitudinal changes in strength and endurance effects of the treatments. Finally, fatigue testing was performed on the contralateral arm first, which may have introduced residual fatigue on the injured arm or reluctance to exert maximal effort during concentric biceps motions.

### Future work

Further research is required to fully understand arm function and recovery following DBT rupture treatments. These results indicate that DBT injury recovery is multifaceted—dependent on the time from injury to surgery, the surgery type, and arm dominance. Future work should involve the development of a prospective multicenter study to understand treatment effects and allow for further subgroup analyses for the various treatment options. Specifically, additional studies should consider the influence of limb dominance on recovery outcomes, as arm dominance appears to significantly affect strength and endurance outcomes. Work is currently underway to determine the feasibility for a multicenter randomized controlled trial to identify optimal surgical decision-making for DBT ruptures.

## Conclusions

Despite functional differences between the healthy and injured arm, operative and non-operative treatments for DBT ruptures demonstrated acceptable long-term functional and PROs. Operative repairs helped to preserve functional strength in supination; however, persistent deficits in endurance may persist following any treatment option. While nonoperative management reported the highest level of dysfunction, there were still satisfactory outcomes and no significant differences to operative repairs. This calls into question the optimal treatment for DBT ruptures and highlights the need for prospective randomized studies to fully investigate the effects of operative and non-operative treatments. Characterizing how functional deficits affect patients' daily activities, return to work capacity, and quality of life will be essential to inform future studies and treatment or rehabilitative strategies.

## Disclaimers:

Funding: Workers' Compensation Board – Alberta: “Evaluating the effectiveness and functional outcomes of treatment options for distal biceps brachii tendon ruptures”. University of Calgary COREF Award: “Distal biceps tendon rupture: A retrospective review of surgical outcomes with prospective functional outcome data”. University of Calgary SPARC Ignite Award: “Identifying optimal treatment strategies for distal biceps brachii tendon ruptures: Surgical or Conservative Treatment?”

Conflicts of interest: The authors, their immediate families, and any research foundations with which they are affiliated have not received any financial payments or other benefits from any commercial entity related to the subject of this article.
