# Supplementary material for: Comparison of functional and patient-reported outcomes following acute, chronic, and nonoperative distal biceps tendon rupture treatments
Source: JSES Rev Rep Tech. 2026 Mar 19;6(3):100728. doi: 10.1016/j.xrrt.2026.100728 (PMC13094442; doi:10.1016/j.xrrt.2026.100728)
Supplement: Appendix Tables Legend [file mmc1.docx]

**Supplementary Material – Table Legends**

**Appendix A**

**Table A-I:** The proprietary Biceps Questionnaire used to assess participant satisfaction levels and motivation for having surgery.

**Appendix B**

**Table B-I:** Occupational data for participants at the time of the strength testing visit, reported as mean (SD)
