# Supplementary material for: Comparison of functional and patient-reported outcomes following acute, chronic, and nonoperative distal biceps tendon rupture treatments
Source: JSES Rev Rep Tech. 2026 Mar 19;6(3):100728. doi: 10.1016/j.xrrt.2026.100728 (PMC13094442; doi:10.1016/j.xrrt.2026.100728)
Supplement: Table A-I [file mmc2.docx]

**Table A-I:** The proprietary Biceps Questionnaire used to assess participant satisfaction levels and motivation for having surgery.

| **Thinking back to why you decided to have surgery to fix your injured biceps: how IMPORTANT were each of the following reasons in your decisions?** | | | | | | | | | |
| --- | --- | --- | --- | --- | --- | --- | --- | --- | --- |
| Select any value from 1-5 for all questions | | 1 (Not important at all) | 2 | | 3 | | 4 | | 5 (Very important) |
| **Improve the appearance of the injured arm** | |  |  | |  | |  | |  |
| **Improve the range of motion of the injured arm** | |  |  | |  | |  | |  |
| **Decrease the pain in the injured arm** | |  |  | |  | |  | |  |
| **Improve strength in the injured arm** | |  |  | |  | |  | |  |
| **A different reason (describe below)** | |  |  | |  | |  | |  |
| **If you selected “A different reason”, please describe it here** | |  | | | | | | | |
| **Please RANK the following reasons from LEAST important to MOST important. You can only select each answer once.** | | | | | | | | | |
| All rows should have a unique 1-5 value. | | 1 (LEAST important) | 2 | | 3 | | 4 | | 5 (MOST important) |
| **Improve appearance of the injured arm** | |  |  | |  | |  | |  |
| **Improve range of motion of the injured arm** | |  |  | |  | |  | |  |
| **Decrease pain in the injured arm** | |  |  | |  | |  | |  |
| **Improve strength in the injured arm** | |  |  | |  | |  | |  |
| **A different reason (describe below) – rank as 1 if not applicable** | |  |  | |  | |  | |  |
| **If you selected “A different reason”, please describe it here. If no “different reason”, please put N/A (not applicable).** | |  | | | | | | | |
| **The following section will ask questions about how satisfied you are with the results of the surgery.**  **How SATISFIED are you with the following?** | | | | | | | | | |
|  | | Not satisfied | | Somewhat satisfied | | | | Satisfied | |
| **Appearance of the repaired arm** | |  | |  | | | |  | |
| **Range of motion of the repaired arm** | |  | |  | | | |  | |
| **Pain in repaired arm** | |  | |  | | | |  | |
| **Strength of the repaired arm** | |  | |  | | | |  | |
| **Have you returned to your pre-injury level of work?** | | YES | | | | NO | | | |
| **Please explain how you are limited (if “No” was selected)** | |  | | | | | | | |
| **Have you returned to your pre-injury level of sport?** | | YES | | | | NO | | | |
| **Please explain how you are limited (if “No” was selected)** | |  | | | | | | | |
| **If you suffered the same injury tomorrow, would you have surgery?** | YES | | | | NO | | | |  |
| **Please explain your choice.** |  | | | | | | | |  |
| **Do you have any other comments about your surgery?** |  | | | | | | | |  |
| **Please comment on how the appearance of your injured arm affected your decision to have surgery, and on the appearance of your arm now.** |  | | | | | | | |  |
