# Supplementary material for: Comparison of functional and patient-reported outcomes following acute, chronic, and nonoperative distal biceps tendon rupture treatments
Source: JSES Rev Rep Tech. 2026 Mar 19;6(3):100728. doi: 10.1016/j.xrrt.2026.100728 (PMC13094442; doi:10.1016/j.xrrt.2026.100728)
Supplement: Table B-I [file mmc3.docx]

**Table B-I:** Occupational data for participants at the time of the strength testing visit, reported as mean (SD)

| **Group** | **Direct Repair** | **Allograft Reconstruction** | **High-Flexion Angle Repair** | **Non-Operative Management** | **Total** |
| --- | --- | --- | --- | --- | --- |
| # of Participants | **25** | **9** | **11** | **15** | **60** |
| Active | 11 | 7 | 5 | 5 | 28 |
| Sedentary | 13 | 2 | 5 | 7 | 27 |
| Retired/Unemployed | 1 | 0 | 1 | 3 | 5 |
